# Psychometric properties of a questionnaire assessing the extent of integration in a problem-based learning curriculum

**DOI:** 10.1186/s12909-025-07165-z

**Published:** 2025-04-17

**Authors:** Marwan F. Abu-Hijleh, Salah Eldin Kassab, Soumaya Allouch, Raja Mahamade Ali, Noor Al-Wattary, Michail Nomikos, Abdel Halim Salem, Mohamed H. Shehata, Reginald P. Sequeira

**Affiliations:** 1 Department of Basic Medical Sciences, College of Medicine, QU Health, Qatar University, P.O. Box: 2713, Doha, Qatar; 2 College of Medicine, Gulf Medical University, P.O. Box: 4184, Ajman, United Arab Emirates; 3 College of Education, Qatar University, P.O. Box: 2713, Doha, Qatar; 4 College of Medicine & Medical Sciences, Arabian Gulf University, P.O. Box: 26671, Manama, Bahrain

**Keywords:** Problem-based learning, Integration, Curriculum design, Evaluation, Questionnaire validation

## Abstract

**Background:**

Integrated curricula reinforce student learning and retention of relevant knowledge. At Qatar University and Arabian Gulf University medical colleges, Problem-Based Learning (PBL) is the principal teaching and learning strategy to implement integrated multidisciplinary system-based curriculum. In addition, other initiatives include integrated assessment, logical organization of teaching and learning methods, as well as combined faculty effort in curriculum planning and delivery. This multicenter study aims to develop and validate a questionnaire for measuring the extent of medical curriculum integration in PBL programs.

**Methods:**

Following an extensive literature review, the content validity of a questionnaire was tested through focus group discussion with subject matter experts (*n* = 20), followed by pilot testing with medical students (*n* = 20). The content-validated questionnaire (31 items) was distributed to students in the two colleges. To examine the construct validity and construct reliability of the questionnaire, exploratory factor analysis (EFA) was conducted with 330 students followed by confirmatory factor analysis (CFA) on 579 students.

**Results:**

EFA supported a four-factor structure of the questionnaire which explained 62% of the variance; however, four items were deleted because of low factor loading (< 0.5) or cross-loading on multiple factors. Further CFA also supported the four-factor structure. Another three items were removed due to high modification indices. The most parsimonious model consisted of 24 items with good fitness indices (χ^2^ = 512.23, df = 236, χ ^2^/df = 2.17, CFI = 0.97, TLI = 0.97, RMSEA = 0.04, SRMR = 0.031, and AIC = 688.22). The four factors were: Planned Curriculum (3 items), Delivered Curriculum (10 items), Assessed Curriculum (4 items) and Learned Curriculum (7 items). The factor loadings of the items ranged from 0.61 to 0.84 with strong correlations between the four latent factors (constructs). The construct reliability of the total questionnaire was 0.97 and the four factors were between 0.94 and 0.97. In addition, there were strong significant correlations between curriculum integration scores and student satisfaction with the curriculum.

**Conclusions:**

The developed questionnaire exhibits good evidence of construct validity and reliability. Further studies required to test the validity of this questionnaire in other settings.

## Background

The term “integration” in educational literature, particularly in the context of medical education, is often used as a buzzword without clear guidance on its development, implementation or evaluation. The concept of an “integrated curriculum” has gained prominence in medical education over the past three decades, yet its meaning and practical application vary widely. Harden et al. (1984) defined integration as *‘the organization of teaching matter to interrelate or unify subjects frequently taught in separate academic courses or departments’* [1]. Decades later, utilizing the spiral model as the ultimate objective, Brauer & Ferguson (2015) defined integrated curriculum as ‘*a fully synchronous*,* trans-disciplinary delivery of information between the foundational sciences and the applied sciences throughout all years of a medical school curriculum’* [2]. It has been shown that integrated medical curricula are more effective than conventional curricula [3, 4]. Learning theories further suggest that adopting an integrated approach to teaching and learning not only enhances students’ comprehension, but also fosters meaningful engagement among adult learners thereby leading to improved knowledge retention [5–8].

Brauer & Ferguson (2015) identified three critical issues for enhancing successful integration in medical education [2]. First, ensuring synchronous presentation of material emphasizes the importance of consistent delivery of integrated knowledge throughout the medical school curriculum. This approach fosters a cohesive understanding of the course material by students and promotes deeper learning. Second, not undermining the importance of basic sciences highlighted by various sources underscores the necessity of integrating foundational knowledge with clinical practice. This integration ensures that students recognize the relevance of basic sciences in their medical education thereby enhancing their clinical reasoning and decision-making skills. Lastly, the use of unified definitions that facilitate a common understanding among stakeholders of the integration process. By establishing clear definitions, educators can align their efforts towards implementing integrated curricula effectively, leading to improved learning outcomes for medical students [2].

The levels of integration in medical curriculum have been explored in various studies [1, 9, 10]. For instance, Shaheen et al. (2022) conducted an exploratory qualitative study and concluded that curriculum integration is inherently inconsistent and does not adhere to a straightforward hierarchical continuum of integration [9]. Interestingly, SPICES model for educational strategies [1] represents integration as a continuum, with full integration at one end and discipline-based teaching at the other. Furthermore, varying levels of integration were discussed, reflecting degrees of interconnectedness and coordination between subjects or disciplines [11]. At the multidisciplinary integration level, subjects are taught separately but efforts are made to align their content and avoid duplication [11]. Moving to interdisciplinary integration, collaboration between disciplines explores shared concepts or themes. Transdisciplinary integration goes beyond disciplinary boundaries, incorporating insights from multiple disciplines into a unified framework [2]. Spiral integration revisits and expands upon previously learned concepts progressively throughout the curriculum [12]. Implementing integration across the entire curriculum demands significant time and effort from both educators and learners. Planning, organizing, and delivering integrated lessons necessitates substantial dedication and effort [13]. The prevailing assumption is that integration should enhance knowledge retention and skill acquisition by iteratively and progressively developing concepts and their applications. However, methods to evaluate whether these objectives were met are infrequently reported, underutilized and poorly understood, thereby limiting sustained success and adoption of integrated curricula.

The multitude of perspectives on integration and its ambiguous nature contribute to the scarcity of instruments, if any, designed to measure the extent and degree of integration in contemporary medical curricula [14]. Admittedly, the term integration is a difficult construct to define. Any meaningful measure of integration in a curriculum begins with an understanding of the many ways to describe it. Despite the challenges of defining integration, there are dimensions to the construct that can provide guidance and boundaries for understanding what constitutes integration [14]. Goldman & Schroth (2012) introduced the “Framework for the Rational Application of Integration as a Guiding Curricular Strategy” which highlights integration as a mean rather than an end goal. The authors stressed that many challenges in implementing integrated curricula stem from overlooking this key distinction [15]. The proposed framework by Goldman & Schroth highlights the need for a systematic approach to integration in order to maximize its advantages and mitigate associated obstacles. Consequently, this framework could serve as the foundation for developing a tool to assess integration levels in problem-based learning (PBL) curricula effectively. PBL is a student-centered teaching approach in which students work in small groups using patient scenarios. PBL encourages students to integrate the knowledge and skills obtained from different disciplines in solving the cases presented to them. Therefore, this study focuses on assessing the degree of integration in medical curricula which implements PBL method.

Based on Goldman & Schroth’s framework, we propose four domains/factors to be covered within the integration questionnaire or tool: 1- Planned curriculum (content), which focuses on the content of the integrated courses, including depth, organization and sequencing of scientific concepts. 2- Delivered curriculum (process), which focuses on the various processes used within the curriculum to ensure proper delivery of concepts. 3- Assessed curriculum (evaluation), which evaluates student performance in terms of content organization, balance, and understanding. 4- Learned curriculum (outcomes), which measures how well the knowledge and skills acquired are applied in medical practice. The objective of this study is to develop and validate an instrument to measure integration as a multi-dimensional construct in PBL medical curricula.

## Methods

### Study context

The population in this study was medical students at the College of Medicine (CMED), Qatar University (QU), Qatar and the College of Medicine and Medical Sciences (CMMS), Arabian Gulf University (AGU), Bahrain. The medical programs at both colleges are six years in duration. Year 1 (phase I) is a foundation year with emphasis on basic medical sciences and general education courses. Pre-clerkship (Phase II) consists of integrated medical and clinical sciences courses arranged by body system. PBL is the main learning strategy in Phase II, and the small-group tutorials are the prevalent educational activity. Clerkship (Phase III) consists of hospital-based rotations in different core clinical specialties. The main difference between the two programs is that the clerkship (Phase III) consists of 2 years at AGU [16] as opposed to 2.5 years at QU [17].

### Development of the questionnaire

The “medical curriculum integration in PBL” questionnaire was designed after we conducted extensive systematic literature review, upon which it was determined that curriculum integration would be best considered as a multidimensional construct [18]. Utilizing Goldman & Schroth’s (2012) framework, this study developed an initial tool to assess integration levels within PBL curricula. The preliminary questionnaire consisted of 53 items operationalized into four dimensions: (1) planned curriculum, (2) delivered curriculum, (3) assessed curriculum, and (4) learned curriculum. A face-to-face focus group discussion was conducted at each institution, involving 10 academics from each site. All participants had over 5 years of experience in medical education, ensuring a high level of expertise. Every focus group discussion began with the signing of a consent form, followed by a 15-minute introduction on the concept of integration by the study authors. A 53-item quantitative questionnaire was then distributed, and a qualitative group discussion took place. Participants in the focus groups first rated each item as essential or non-essential based on the degree of concordance between the item and the intended construct. In addition, participants were asked to comment on the degree of clarity of each item and suggest any modifications if needed. Participants’ ratings of the questionnaire items were used to calculate the content validity index. The focus group discussion led the experts to agree on retaining 33 items, with only minor or no modifications. Meanwhile, 20 items were discarded based on the content validity index (below 75%), clarity issues, and expert suggestions for deletion or incorporation of new items. The 33-items questionnaire was then assessed by all authors in two consecutive rounds of online review meetings and consensus was achieved through discussion. The questionnaire was then pilot-tested with a group of 20 medical students, evenly divided between the two institutions (*n* = 10 from each). The pilot testing aimed to assess the suitability, clarity, and relevance of the questionnaire items. Based on the feedback provided by the students, further modifications were made to enhance the questionnaire’s quality and usability. Additionally, two items were removed as they were deemed unsuitable or redundant according to the students’ input.

### Study design and sampling

This study used a cross-sectional correlation design with convenient sampling of the population of medical students. The paper-based questionnaire was completed by 579 students (response rate = 55.99%). According to published guidelines, this sample is appropriate for achieving replicable factor analysis outcomes. Sample sizes of 300 or more are generally sufficient, 150–200 may suffice under specific conditions, and sizes below 100 are typically inadequate [19]. Based on their subjective experience, students were asked to rate the extent to which each item is applicable to their PBL curriculum. Items measuring curriculum integration were rated on a Likert-like scale of 1 to 4 (1 = to no extent, 2 = to a low extent, 3 = to a moderate extent, and 4 = to a large extent). The 4-point Likert-scale was chosen to provide definitive ratings and avoid neutral responses. Additionally, these responses were used in the factor analysis of the questionnaire, which aimed at excluding items that fails to measure curriculum integration.

### Statistical analysis

In this study, a sample of 330 students were included in an exploratory factor analysis (EFA) to assess factors underlying the curriculum integration questionnaire. Principal Component Analysis (PCA) was used to extract emerging factors employing Kaiser Rule with an eigenvalue criterion greater than 1 for factor determination. The sampling adequacy was verified through Bartlett’s Test of Sphericity and the Kaiser-Meyer-Olkin (KMO) Test. Factor retention decisions were informed by assessments of scree plots, factor loadings, and theoretical coherence [20]. Interpretation of the factors, considering communalities and cross-loadings, provided insights into the underlying constructs of curriculum integration.

Confirmatory factor analysis (CFA) was then applied on data from 579 students using maximum likelihood estimation to examine the degree of fitness between the measurement model and the underlying structural model (latent factors representing curriculum integration). Multivariate normality was assessed using Amos by examining Mardia’s normalized estimate of multivariate kurtosis. Missing data which comprised 6 samples were addressed using listwise deletion. This approach is considered appropriate when the proportion of missing data is 5% or less, as it minimizes bias while maintaining the integrity of the analysis. Various indices were employed to evaluate the congruence between the measurement model and the underlying theoretical framework. The Comparative Fit Index (CFI) serves to measure the comprehensive performance of the examined model in comparison to a baseline (independence) model. A CFI value of ≥ 0.95 is commonly used as a criterion for model acceptance, indicating that the specified model can replicate 95% of the covariation within the data [21, 22]. The Chi-Square (χ²) test indicates the extent of concordance between the implied and observed covariance matrices. An insignificant χ² or a χ²/df ratio < 5 signifies a satisfactory fit for the model [23]. The Root Mean Square Error of Approximation (RMSEA) delineates the average disparity between the observed and predicted covariances, with a value ≤ 0.06 denoting an adequate model fit [22]. The Standardized Root Mean Square Residual (SRMR) is defined as the standardized mean deviation between the observed correlation matrix and the model-implied correlation matrix [22]. A value < 0.06 is indicative of an acceptable fit, albeit influenced by sample size and model complexity. The Tucker Lewis Index (TLI) or Non-normed Fit Index (NNFI) provides an additional metric commonly employed to evaluate model fitness with an acceptable value of 0.95 [22]. Finally, the Akaike Information Criterion (AIC) facilitates the comparison of various potential models based on their capacity to leverage all available data. Lower AIC values signify superior fit. Optimal model selection is contingent upon consideration of the different fitness indices.

### Construct reliability (CR)

Composite (or construct) reliability is a measure of internal consistency in the observed indicators that load on a latent variable (construct). Calculation of construct reliability as applied in this study has been previously reported [24]. The formula for calculating construct reliability is as follows:

$$\:CR=\frac{{\left(\sum\:{\lambda\:}_{i}\right)}^{2}}{{\left(\sum\:{\lambda\:}_{i}\right)}^{2}+\left(\sum\:{\epsilon\:}_{i}\right)}$$

where λ (lambda) is the standardized factor loading for item *I*, and ε is the respective error variance for item *i*. The error variance (ε) is estimated based on the value of the standardized loading (λ).

### Predictive validity

Correlations were computed between the four integration factors and student satisfaction with the integrated curriculum by using *Pearson’s correlation coefficient*.

## Results

Table 1 shows the demographic characteristics of medical students involved in the study. The ages of most student ranged from 18 to 27 years and 61.3% of students were females. The study sample included students from year 3, 4, 5, and 6 of the programs. Earlier years were not involved because the process of problem-based learning starts from year 2 onwards.

This study describes the iterative process of refining a 53-item questionnaire into a final 24-item questionnaire. The instrument was reviewed by an expert panel from QU and AGU and was found to have a satisfactory content validity. The instrument was found to have good face validity in pilot testing, and all students found the items of the questionnaire to be clear and logically flowing. The resultant items were then subjected to factor analysis to assess the construct validity of the instrument. The final instrument consists of 24 items with 4-point Likert scale response options ranging from 1 to 4 (1 = to no extent, 2 = to a low extent, 3 = to a moderate extent, and 4 = to a large extent).

The EFA of the “Curriculum Integration in PBL Questionnaire” data yielded four distinct factors identified as planned curriculum, delivered curriculum, assessed curriculum, and learned curriculum (outcomes of integration). These factors collectively accounted for a substantial 62.8% of the variance within the dataset. The KMO test was 0.96 and Barlett’s test was significant (*P* = 0.000) indicating excellent sampling adequacy and factorability of the variables [20]. Moreover, correlations between items within each factor were substantial, providing further support for the coherence and relevance of the identified components. By examining the rotated matrix, two items were deleted due to loadings < 0.5. These items were “curriculum is responsive to feedback from stakeholders”, and “Foundation courses enhance curriculum integration”. Another two items were deleted due to significant cross loading > 0.4 on more than one factor. These items were “PBL tutorials enhance curriculum integration”, and “PBL cases integrate basic sciences, clinical sciences, and population health”.

**Fig. 1:**
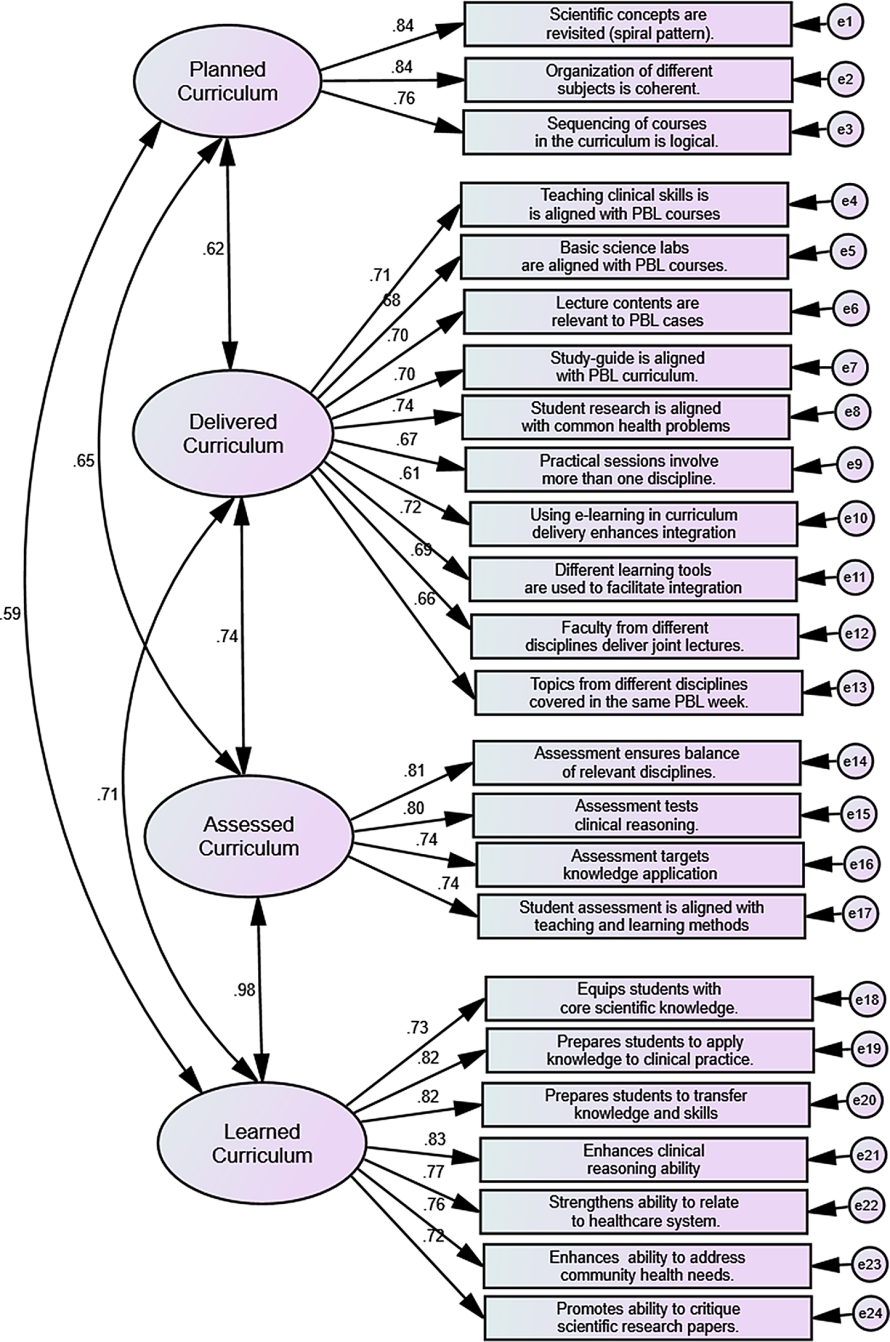
Confirmatory factor analysis of the “curriculum integration in PBL questionnaire”. Data are collected from medical students (*N* = 579)

As shown in Fig. 1, confirmatory factor analysis (CFA) was conducted using the 31-item model of the questionnaire. The fitness indices of the 31-item questionnaire were as follows: χ^2^ = 1697.96, df = 399, χ ^2^/df = 4.26, CFI = 0.90, TLI = 0.89, RMSEA = 0.08, SRMR = 0.076, and AIC = 1889.96.

It was evident that the 31-item measurement model did not satisfactorily fit the theoretical model. According to the EFA results which recommended deletion of four items, the CFA analysis was then conducted using the 27-item questionnaire. The results of the analysis indicated improved fitness; however, the indices were still not satisfactory. The results of this round of analysis were as follows: χ^2^ = 1114.89, df = 292, χ ^2^/df = 3.92, CFI = 0.93, TLI = 0.92, RMSEA = 0.07, SRMR = 0.054, and AIC = 1284.89. As a result, the modification indices report were examined, resulting in the removal of another 3 items due to high modification indices. These items were “PBL allows integration across basic sciences”, “The curriculum enhances student ability to write scientific research papers”, and “Primary healthcare placements/visits provide a learning environment to integrate knowledge and skills”. Following this change, the 24-item questionnaire model was the most parsimonious and demonstrated good fit with the original theoretical analyses. Therefore, further analyses were conducted using this model. The fitness indices of the 24-item questionnaire were: χ^2^ = 512.23, df = 236, χ ^2^/df = 2.17, CFI = 0.97, TLI = 0.97, RMSEA = 0.04, SRMR = 0.031, and AIC = 688.22. Results of the goodness of fit for the models mentioned above are presented in Table 2.

Figure 1 shows factor loadings for each of the items on the four latent factors. The four factors were (1) planned curriculum (3 items), (2) delivered curriculum (10 items), (3) assessed curriculum (4 items), and (4) learned curriculum (7 items). The factor loadings of the items ranged from 0.61 to 0.84 with strong correlations between the four latent factors (constructs). Table 3 describes final questionnaire items (24 items) and the four factors or constructs identified through factor analysis.

### Construct reliability

The data demonstrated excellent construct reliability for the four factors underlying the curriculum integration questionnaire. The construct reliability of the four latent factors was 0.97 for “planned curriculum”, 0.96 for “delivered curriculum”, 0.97 for “assessed curriculum”, and 0.94 for “learned curriculum”.

### Predictive validity

There were strong positive correlations between student satisfaction with the curriculum and the extent of perceived curriculum integration by students. The correlations between student satisfaction and “planned curriculum” was 0.57 (*P* = 0.000), “delivered curriculum” was 0.54 (*P* = 0.000), “assessed curriculum” was 0.72 (*P* = 0.000), and “learned curriculum” was 0.74 (*P* = 0.000).

**Table 1:** Demographic characteristics of medical students at QU and AGU

Characteristic	Qatar University	Arabian Gulf University
**Respondent**	186 (61.3)	365 (49.9)
Gender*		
Male	60 (32.3)	106 (29.0)
Female	114 (61.3)	259 (71.0)
Year of study*		
Year 3	62 (33.3)	136 (37.3)
Year 4	48 (25.8)	47 (12.9)
Year 5	28 (15.1)	92 (25.2)
Year 6	36 (19.35)	90 (24.7)
**Age**		
18–22	126 (67.7)	239 (65.5)
23–27	47 (25.3)	125 (34.2)
28–32	1 (0.5)	1 (0.3)

*Missing data for this variable

**Table 2:** Fitness indices for measurement models of the students’ perceptions of curriculum integration in PBL questionnaire (*N* = 579)

	Measurement models	χ ^2^	df	χ ^2^/df	CFI	TLI	RMSEA (90% C.I.)	SRMR	AIC
1	Four latent factors − 31 items	1697.96	399	4.26	0.90	0.89	0.08(0.07–0.10)	0.076	1889.96
2	Four latent factors − 27 items	1114.89	292	3.82	0.93	0.92	0.07(0.07–0.09)	0.054	1284.89
3	Four latent factors − 24 items	512.23	236	2.17	0.97	0.97	0.04(0.04–0.05)	0.031	688.22

**Table 3:** Final factors with questionnaire items

**Factor 1: Planned curriculum (content)**	
1	Scientific concepts are revisited in depth and breadth in the curriculum as students’ progress (spiral pattern).
2	The organization of different subjects within each unit (course) is coherent.
3	The sequencing of units (courses) in the curriculum is logical.
**Factor 2: Delivered curriculum (process)**	
4	Teaching of clinical skills is aligned with PBL units (courses).
5	Basic science labs are aligned with PBL units (courses).
6	Resource session/lecture content is relevant to PBL cases.
7	Student study-guide (unit booklet) is aligned with PBL curriculum.
8	Student research is aligned with common health problems in the curriculum.
9	Practical/lab sessions involve more than one discipline.
10	Using e-learning in curriculum delivery (teaching and assessment) enhances integration.
11	Different learning tools are used to facilitate integration (such as concept mapping, mind mapping etc.).
12	Faculty from different disciplines participate in delivering joint resource sessions/lectures.
13	Topics from different disciplines are covered in the same PBL week.
**Factor 3: Assessed curriculum (evaluation)**	
14	Student assessment ensures balanced representation of relevant disciplines.
15	Student assessment includes questions that test clinical reasoning.
16	Student assessment targets knowledge application rather than recall.
17	Student assessment is aligned with the teaching and learning methods.
**Factor 4: Learned curriculum (outcomes)**	
18	The curriculum equips students with core scientific knowledge.
19	The curriculum prepares students to apply theoretical knowledge to clinical practice.
20	The curriculum prepares students to transfer gained knowledge and skills to other closely related clinical problems.
21	The curriculum enhances clinical reasoning ability (problem-solving) of students.
22	The curriculum strengthens student ability to relate population health issues to healthcare systems.
23	The curriculum enhances student ability to address community health needs.
24	The curriculum promotes student ability to critique scientific research papers.

## Discussion

The purpose of this study was to develop, evaluate and validate a tool for medical educators and researchers to assess the degree of integration in a PBL medical curriculum. The developed instrument was evaluated for evidence of content, construct, and predictive validity measures. The integrated curriculum in its most comprehensive definition is ‘a fully synchronous, trans-disciplinary delivery of information between the foundational sciences and the applied sciences throughout all years of a medical school curriculum’ [2]. This definition by Brauer & Ferguson (2015) takes into consideration the organizational approach of planning for an integrated curriculum before it is implemented. Other definitions focus on a higher level of integration wherein knowledge from various disciplines converges within the learners’ cognition. This higher-level integration represents the desired result of integrated curricula [25].

Our team developed the instrument following a systematic review that revealed a lack of comprehensive tools for evaluating curriculum integration across all dimensions. Although 22 instruments were identified in this review, only 3 focused on evaluating the curriculum’s integration levels (data is under consideration for publication). Similar to our study, The first instrument is “Howard’s integrated curriculum evaluation instrument” which assesses the extent of vertical and horizontal integration according to Fogarty’s ten levels of integration [26, 27]. This instrument is qualitative; participants are given a definition for each level of integration based on which they rate their curriculum. However, the instrument’s qualitative nature renders it susceptible to bias as individuals may interpret the meaning of each level differently. Additionally, this instrument provides broad definitions of each category without a deeper inquiry about the content of the integrated curriculum, planning, or modes of delivering the integrated content. The other two instruments are the “integration ladder questionnaire”, developed based on Harden’s integration ladder, and “the session integration tool (SIT)” [28, 29]. Both instruments are useful in determining integration level; however, the SIT’s scope is narrow as it focuses on integration of educational content within a session.

Goldman & Schroth (2012) emphasize that curriculum integration should be approached as a subset of general curriculum development decisions and propose a three-level framework for applying integration as a guiding curricular strategy: program-level, course-level, and session level [15]. Program level encompasses integration forms, purpose, dimensions, environment, and coherence. Course level is focused on types of integration used to achieve the programs’ outcomes, and its implication on faculty and scheduling. Session level integration is related to the preparatory work, linking of meaning, engagement, and transfer of knowledge. Based on this broad framework and following a review of the literature, we designed the initial tool and questionnaire that comprised 53 items operationalized into four domains: planned curriculum, delivered curriculum, assessed curriculum, and learned curriculum. The developed instrument is more comprehensive as it assesses integration on a program level. It also provides an extensive evaluation of the level of integration on a horizontal, vertical and spiral level. Notably, the majority of integration instruments found in the literature overlooked the spiral integration within a curriculum [18]. Instead, they focus primarily on measuring horizontal and vertical integration particularly between basic and clinical sciences. Spiral integration, which involves a combination of both horizontal and vertical integration between different disciplines across time, is the most complete form of integration [30]. It reinforces knowledge by naturally progressing topics from simplicity to complexity throughout a curriculum, effectively breaking down barriers between disciplines [30]. The integration of basic and clinical sciences alone not enough, it is also necessary to emphasize the importance of humanism and health population sciences in medicine [31]. This is an advantage of the current study instrument, which assesses integration across different disciplines including population health.

Factor analysis of the curriculum integration instrument resulted in identification of four factors (constructs) and the exclusion of seven items. The remaining 24 items were distributed across the constructs which are: (1) Planned, (2) Delivered, (3) Assessed, and (4) Learned curricula. The results of the factor analysis confirmed the multidimensionality of the integration assessment instrument. Interestingly, these four constructs seem to be consistent with the categories of the “context, input, process and product (CIPP)” model [32]. The first factor of the instrument; namely planned curriculum, assessing how the curriculum was designed to ensure the fulfilment of integration requirements would best fit under the input component of the CIPP [33]. On the other hand, factors 2 (delivered curriculum) and 3 (assessed curriculum) are focused on whether curriculum delivery and student assessment are consistent with the process element of the model. The fourth factor (learned curriculum) assesses whether student-learning outcomes are representative of the product component. This finding suggests that the developed instrument is thorough, enabling the assessment of curriculum integration from the initial planning phase through content delivery and student evaluation, and concluding with the outcomes of integration as well as the impact on student knowledge application in practice. The factor structure is also consistent with the four dimensions developed according to the adopted conceptual framework (i.e. planned curriculum, integration process, integrated assessment, and integration outcome).

Assessing the content of the curriculum, its organization, and the extent of its contribution to integration of knowledge and skills is an important component of integrated curriculum evaluation. Therefore, items under factor 1 of the questionnaire play an important role in assessing this aspect of the curriculum [34]. Designing integrated assessments is an essential component of an integrated curriculum which should be done simultaneously with integrated curriculum planning and implementation. Integrated curricula which lack integrated students’ assessments are likely to be ineffective and fail in achieving the desired outcomes of integration [35]. Assessments significantly impact students’ learning by shaping their perceptions of which key content areas to focus on, their learning approaches, and their expectations of what will be included in the assessment [36]. Therefore, content, means, and location of the assessments influence medical students’ learning. This learning is also shaped by the feedback received after completing the assessments.

The results of this study reveal that students’ satisfaction with their curriculum was positively associated with their perceived integration level. This suggests that students who view the curriculum as integrated are also satisfied with the curriculum overall; a finding which reflects that students have a positive view of curriculum integration. This is consistent with the findings of other studies that report medical student satisfaction with integrated curricula [10, 37–40]. Medical students often express their preference for integrated curricula over traditional ones [41]. Similarly, students’ satisfaction in this study correlates positively with all factors of the instrument, and more prominently with factors 3 and 4 (assessed curriculum and learned outcomes). Medical students also express their satisfaction with integrated curriculum’s content and teaching methods [39]. The effectiveness of an integrated curriculum hinges on achieving the program’s goals. This includes equipping students with skills to connect and integrate knowledge from different courses, disciplines and learning methods, and enabling practical application [33]. Last factor of the integration instrument assesses this aspect as part of the curriculum evaluation.

This study has several strengths. First, it evaluates the psychometric properties of a comprehensive integration level assessment instrument, employing various measures such as content, construct, and predictive validity. Second, it is multi-institutional and involves medical students from two different countries. Third, the focus on evaluating an integrated PBL curriculum adds strength to the study, given PBL’s widespread adoption in medical education. Additionally, the instrument assesses all three levels of integration (vertical, horizontal, and spiral) further enhancing its robustness. However, the self-reported nature of measuring curriculum integration in this study could be biased by perceptions of students rather than actual measurement of curriculum integration. In addition, measuring the integration using students only and not involving other stakeholders such as faculty members could limit the generalizability of the study findings. Therefore, future utilization of this instrument in a broader sample encompassing more medical students and faculty will be required. In addition, establishing other sources of validity evidence such as correlations with student achievement in integrated assessments is advised to further refine the psychometric properties of the study instrument.

## Conclusions

This study describes the development and validation of an instrument assessing the degree of integration in medical PBL-based curriculum. The developed instrument was found to have good construct validity, with factor analysis identifying four constructs: (1) planned curriculum, (2) delivered curriculum, (3) assessed curriculum, and (4) learned curriculum. The instrument was also found to have good predictive validity such that students’ perception of integration level significantly predicted students’ satisfaction with the overall curriculum. The integration evaluation instrument developed in this study has promising potential and its application in a larger and more diverse sample is encouraged.

## Data Availability

Data is provided within the manuscript or supplementary information files.

## Abbreviations

PBL: Problem Based learning
SPICES: Student-centered, Problem-based, Integrated, Community-based, Elective, Systematic
CMED: College of Medicine
QU: Qatar University
CMMS: College of Medicine and Medical Sciences
AGU: Arabian Gulf University
PCA: Principal Component Analysis
EFA: Exploratory Factor Analysis
KMO: Kaiser-Meyer-Olkin
CFI: Comparative Fit Index
RMSEA: Root Mean Square Error of Approximation
SRMR: Standardized Root Mean Square Residual
TLI: Tucker Lewis Index
NNFI: Non-normed Fit Index
AIC: Akaike Information Criterion
CR: Construct reliability
CFA: Confirmatory Factor Analysis
SIT: Session Integration Tool
CIPP: Context, Input, Process and Product
